# Assessing Awareness Level about Warning Signs of Cancer and its Determinants in an Iranian General Population

**DOI:** 10.3329/jhpn.v29i6.9904

**Published:** 2011-12

**Authors:** Awat Feizi, Anoshirvan Kazemnejad, Mohsen Hosseini, Zohreh Parsa-yekta, Jamshid Jamali

**Affiliations:** ^1^Department of Biostatistics and Epidemiology, School of Health, Isfahan University of Medical Sciences, Isfahan, Iran; ^2^Department of Biostatistics, School of Medical Sciences, Tarbiat Modares University, Iran; ^3^Department of Nursing, Faculty of Nursing and Midwifery, Tehran University of Medical Sciences, Iran; ^4^Vice-chanellery for Research, Mashhad University of Medical Sciences, Iran

**Keywords:** Awareness, Cancer, Cross-sectional studies, Health education, Neoplasms, Public education, Signs and symptoms, Iran

## Abstract

The present study was aimed at investigating the awareness level about warning signs of cancer and its determinants in an Iranian general population. This cross-sectional interview-based survey investigated 2,500 people aged 18 years and over, as a representative sample of Tehran population. Latent class regression was applied for analyzing data. A small (18.8%) proportion of the respondents had high level of knowledge, and 54.5% had moderate awareness, and 26.7% had low level of awareness. Most effective predictors for awareness were educational attainment, sex, and marital status. The findings suggest that the overall level of knowledge about warning signs of cancer among the public is low, particularly about some specific signs. Accordingly, educational and intervention programmes, with special attention placed on particular at-risk populations, to increase awareness about the disease leading to its early diagnosis are needed.

## INTRODUCTION

Cancer is the third cause of mortality in developing countries (1). In 2007, more than 12.3 million people were affected by different kinds of cancers; more than 7.6 million died of the disease (around 13% worldwide), in which more than 70% of all deaths due to cancer occurred in developing countries; and 84 million people will die in the next 10 years, if action is not taken (1). The estimated number of new cases may rise from 12.3 million in 2007 to 16 million in 2020, and approximately 60% of all these new cases may occur in low-and middle-income countries (2).

In Iran, cancer is the third major cause of death (11.8%), and it is estimated to increase to 13.4% by 2030 (2-3). Annually, 30,000 Iranians die of cancer (3). It is estimated that more than 70,000 new cases of cancer may be detected in the country, and the incidence of cancer in the next decade will rise due to an increase in the number of the elderly people of the country (3).

Awareness of public about warning signs of cancer in relation to early detection and prevention has been surveyed in a few countries only, and results showed poor knowledge among them (4-8). Other studies focusing on specific cancers are limited in terms of sample-size or composition, and most of these studies were conducted in purposive samples or clinical settings, e.g. restricted age, sex, or patient groups (9-12).

In Iran, there is virtually no information on early detection and prevention knowledge of cancer among the general population. We decided to explore the level of knowledge about warning signs of cancer and its determinants among the generalpopulation, aged 18 years and over, in Tehran, Iran.

To the best of our knowledge, this study is not only the first large-scale population-based study in a developing country but it also can be considered one of few studies in the world, which seeks public awareness of warning signs of cancer. Our research, compared to most other studies in this area, is not a pure descriptive study, it rather uses a comprehensive statistical modelling framework.

## MATERIALS AND METHODS

This exploratory and correlational cross-sectional study was conducted among the general population living in Tehran, Iran. Sample selection was performed with a probabilistic multi-stage stratified cluster sampling. In total, 2,500 people aged 18 years and over were interviewed. All the participants were informed about the study and signed a written consent form.

In the present study, knowledge of nine possible warning signs of cancer that were reported either in the European Code Against Cancer or by the major cancer organizations was assessed. These warning signs include: (a) changes in bowel or bladder habits; (b) a sore that does not heal; (c) unusual bleeding or discharge; (d) thickening or lump in the breast or elsewhere; (e) obvious change in a wart or mole; (f) nagging cough or hoarseness; (g) unexplained loss of weight; (h) difficulty in swallowing, and (i) indigestion.

The aggregate level of knowledge about all warning signs of cancer was considered a latent construct and was evaluated based on the dichotomous variables through latent class regression analysis (13). This model also provides the possibility for evaluating the effects of correlated determinant of knowledge level.

All descriptive and analytical statistical methods in the study were performed using the R free software (version 2.11.1).

## RESULTS

The most important descriptive findings regarding the demographic characteristics of the respondents (n=2,508) were as follows: 52.9% were women; 37.2% were single; 42.3% had high school education; 30.8% had university attainments; 17.7% had a family history of cancer; 26.6% were smokers; and 10.4% were alcohol drinkers. More details about the demographic characteristics of the study respondents were reported elsewhere (14).

**Table 1.** Prevalence of correct knowledge about specific warning signs of cancer

Warning sign	Total		Men		Women	
	%	95% CI	%	95% CI	%	95% CI
Change in bowel or bladder habits	32.70	32.68-32.72	31.50	31.47-31.53	33.80	33.77-33.83
A sore that does not heal	43.20	43.18-43.22	40.40	40.37-40.43	45.60	45.57-45.63
Unusual bleeding or discharge	47.70	47.68-47.72	43.00	42.97-43.03	51.40	51.37-51.43
Thickening or lump in breast and other organs	67.40	67.38-67.42	56.90	56.87-56.93	76.80	76.78-76.82
Difficultly in swallowing	33.60	33.58-33.62	30.20	30.17-30.23	36.50	36.47-36.53
Indigestion	37.80	37.78-37.82	35.00	34.97-35.03	40.30	40.27-40.33
Change in a wart or mole	49.80	49.78-49.82	44.70	44.67-44.73	54.50	54.47-54.53
Nagging cough or hoarseness	42.40	42.38-42.42	38.10	38.07-38.13	46.20	46.17-46.23
Unexplained loss in weight	59.20	59.18-59.22	56.10	56.07-56.13	61.90	61.87-61.93

CI=Confidence interval

**Table 2.** Class-specific level of awareness about warning signs of cancer and the size of classes

Warning sign	Class 1 Level of awareness-high		Class 2 Level of awareness-moderate		Class 3 Level of awareness-poor	
	Yes (%)	No (%)	Yes (%)	No (%)	Yes (%)	No (%)
Change in bowel or bladder habits	85.83	14.17	28.06	71.94	4.73	95.27
A sore that does not heal	95.48	4.52	44.84	55.16	2.82	97.18
Unusual bleeding or discharge	98.22	1.78	52.18	47.82	1.95	98.05
Thickening or lump in breast and other organs	99.86	0.14	81.74	18.26	15.36	84.64
Difficulty in swallowing	95.00	5.00	28.35	71.65	0.86	99.14
Indigestion	94.49	5.51	34.79	65.21	3.96	96.04
Change in a wart or mole	98.20	1.80	56.09	43.91	2.98	97.02
Nagging cough or hoarseness	93.00	7.00	44.08	55.92	3.27	96.73
Unexplained weight loss	94.66	5.34	70.69	29.31	10.61	89.39
Class size (%)	18.82		54.49		26.69	

Table 1 presents the prevalence of correct knowledge on warning signs of cancer, with 95% confidence intervals for all the study participants—men and women. The table shows that, on a sign-by-sign basis, women were more aware than men (on average, 49.66% vs 41.76%)

Table 2 shows the percentages of the correct answers to the questions on specific warning signs of cancer in each constructed classes. Class 1 included the individuals with higher knowledge level (class size: 18.8%), Class 3 included the individuals with poor awareness level (26.7%), and Class 2 included the individuals with mixed situation in terms of the awareness level (54.5%). The constructed classes (latent classes) play the role of categories of the dependent variable. Therefore, similar to multinomial logistic regression, Class 1 (high knowledge) was selected as a reference class.

Table 3 contains the class-specific estimates of regression coefficients. The significant effective predictors of awareness level about warning signs, in order of importance, were level of education, gender, and marital status. Although other studied factors, such as age, family history, and lifestyle behaviours (smoking and alcohol drinking) were significant, they had a weak discriminative role.

**DISCUSSION**

The results revealed that, in general, the level of knowledge about warning signs of cancer among the studied sample was low. Our findings are consistent with those of a few studies in developing countries (6-7). However, the level of awareness in a developed country is slightly higher (4,5,8). Such a difference, in general, can be attributed to the social inequalities between developing and developed countries (7).

Assessment of the effective factors on the knowledge level about warning signs of cancer indicated that the strongest predictor was the level of education. Our findings are consistent with findings of other studies (4-6,9,14-17). It is possible to infer that people having high levels of educational attainment are in a better position to be able to attend to the health protective issues.

The results of our research showed that men were significantly less likely than women to be aware of the early warning signs of cancer. These findings are consistent with those of other studies evaluating the effect of gender on knowledge of cancer (7,8,14-17). A possible explanation for this difference may lie with women's greater familiarity and use of primary healthcare services through, for example, participation in mass health programmes and their responsibility for their children's healthcare and interest in health within families (18).

**Table 3.** Estimated class-specific covariates coefficients and related z-statistics and p values

Variable	Coefficients	z value (p value)	Odds ratio	95% CL
Class 2 (ref=Class 1)				
Age	-0.02	-2.79 (0.005)	0.97	0.96-0.98
Sex (ref=women)	0.31	4.6 7(0.025)	1.36	1.04-0.77
Marital status (ref=unmarried)	-0.62	-3.73 (0.00)	0.54	0.39-0.75
Level of education	-0.76	-8.56 (0.00)	0.47	0.39-0.55
Family history (ref=without)	-0.28	-1.70 (0.089)	0.76	0.55-1.04
Smoking (ref=non-smoker)	0.16	0.98 (0.33)	1.18	0.85-1.63
Alcohol drinking (Ref=non-drinker)	0.21	1.97 (0.048)	1.23	1.03-1.53
Class 3 (ref=Class 1)				
Age	-0.002	-0.18 (0.85)	0.98	0.99-1.00
Sex (ref=women)	0.91	-5.71 (0.00)	2.47	1.81-3.37
Marital status (ref=unmarried)	-0.69	-3.43 (0.001)	0.50	0.34-0.75
Level of education	-1.21	-11.62 (0.00)	0.30	0.24-0.37
Family history (ref=without)	-0.35	-1.49 (0.091)	0.70	0.44-1.12
Smoking (ref=non-smoker)	-0.57	-4.83 (0.00)	0.57	0.39-0.82
Alcohol drinking (ref=non-drinker)	0.29	2.69 (0.007)	1.33	1.11-1.55

CI=Confidence interval;

ref=Reference category

The results showed that the married people, particularly women, were more concerned about their health because of their responsibilities to the families. Hence, they had a higher level of awareness compared to the single ones. Our findings are consistent with those of Brunswick *et al*. and Evans *et al.* (5,18).

Previous studies on adult population in developed countries have shown that the middle-aged people recognized more signs than those who were younger or older (5,19). However, in our study this factor provided weak discriminative effect (see the OR values).

It was expected that those with a family history of cancer (they may be more motivated to adopt preventive behaviours) were particularly likely to acknowledge the potential warning signs of cancer (20). However, the findings of our study indicated that the family history had little influence on increasing the level of awareness.

### Conclusions

These data may be considered the first step in the development of an intervention based on empirical findings that will identify areas for public education and intervention efforts as an important component of prevention of the disease. Such educational and intervention programmes should be culture-sensitive and accessible to all individuals, with special attention placed on reaching the populations of the highest risk to increase awareness about the disease leading to its early diagnosis. As a national cancer strategy, public education combined with the use of cancer-screening technology, focused on high-risk populations, is a cost-effective approach.

## ACKNOWLEDGEMENTS

The study was financially supported (in part) by the Tehran University of Medical Sciences; however, all work was initiated and analyzed by the investigators.
